# Circular RNA_ANKIB1 accelerates chemo-resistance of osteosarcoma via binding microRNA-26b-5p and modulating enhancer of zeste homolog 2

**DOI:** 10.1080/21655979.2022.2037869

**Published:** 2022-03-09

**Authors:** JinShan Tang, Gang Duan, YunQing Wang, Bin Wang, WenBo Li, ZiQiang Zhu

**Affiliations:** a Department Orthopedics, Huai’an Second People’s Hospital, Huai’an City, JiangSu Province, China; b Department Orthopedics, Huaian Hospital Affiliated to Xuzhou Medical University, Huai’an City, Jiangsu, China; c Department Orthopedics, The Second Affiliated Hospital of Xuzhou Medical University, Xuzhou City, JiangSu Province, China

**Keywords:** Circular RNA_ANKIB1, MicroRNA-26b-5p, enhancer of zeste homolog 2, osteosarcoma, doxorubicin resistance

## Abstract

Osteosarcoma is a common bone malignancy in children and adolescents. Chemotherapeutic drug resistance is the major factor impacting the surgical outcome and prognosis of patients with osteosarcoma. This investigation assessed the role and mechanism of circular RNA_ANKIB1 in the development of osteosarcoma. The circular RNA (circ) _ANKIB1, microRNA (miR)-26b-5p, enhancer of zeste homolog 2 (EZH2) expression in OS samples was investigated through RT-qPCR. The EZH2, multidrug resistance protein 1 (MRP1), P-gp, and lipoprotein receptor-related protein (LRP) protein expressions were analyzed through western blot. The association between circ_ANKIB1 and the occurrence of clinic-pathological features in OS patients was assessed; the circular features of circ_ANKIB1 were analyzed. The hFOB1.19, KHOS, U2-OS OS cells were used to study the semi-inhibitory concentration IC50 of Doxorubicin (DXR)-resistant cells, clone formation, invasion, and apoptosis. The luciferase assay was used to study the binding of circ-ANKIB1 with miR-26b-5p and the targeting of miR-26b-5p with EZH2. In *vivo* experiments were performed via subcutaneous tumorigenic experiments. MiR-26b-5p in OS tissues and cells and DXR-resistant OS tissues and cells was silenced while circ_ANKIB1 and EZH2 were elevated. Circ_ANKIB1 silencing elevated miR-26b-5p, repressed EZH2, MRP1, P-gp, LRP, IC50, and elevated OS advancement. Circ_ANKIB1 bind miR-26b-5p. Reduced miR-26b-5p revered the influence of silencing circ_ANKIB1 on DXR resistant OS cells. MiR-26b-5p targeted EZH2, and EZH2 elevation reversed the impact of increasing miR-26b-5p on DXR resistant cells. Circ_ANKIB1 silencing suppressed DXR-resistant OS cells in *vivo*. In conclusion, Circ_ANKIB1 binds miR-26b-5p and modulates EZH2 to accelerate the chemo-resistance of osteosarcoma.

## Graphical abstract

**Figure:**
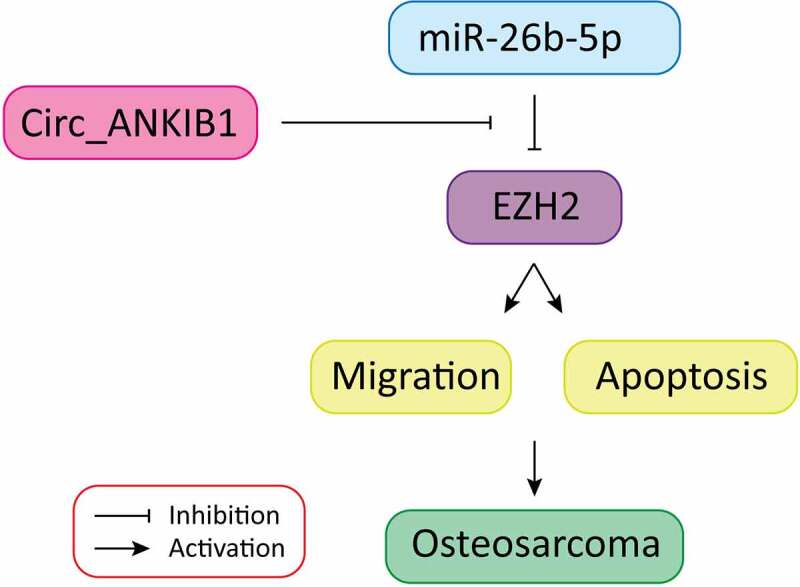

## 1 Introduction

Osteosarcoma (OS) is a prevalent bone malignancy in children and adolescents [1]. As a mesenchymal tumor, OS tends to emerge in the metaphysis of long bones with abundant blood supply and fast growth, covering the proximal humerus, distal femur, or proximal tibia [2]. The OS has a rapid progression leading to high morbidity and mortality [3]. The overall survival rate of OS has been significantly increased in the past few decades due to advances in surgery and chemotherapy [4]. Chemotherapeutic drugs have been adopted for the treatment of OS since the 1970s, with doxorubicin (DXR) being the primary chemotherapy regimen [5]. Nevertheless, chemoresistance is the major adverse occurrence influencing the efficacy of chemotherapy drugs and reducing the survival rate of patients with OS [6]. Consequently, there is a need to understand the molecular mechanisms leading to chemotherapy resistance, to improve the prognosis of OS.

Circular RNA (circRNA) is a form of non-coding RNA discovered a few decades ago. According to reports, circ RNA has a crucial role in the occurrence and progression of tumors [7]. According to reports, the competitive endogenous RNA (ceRNA) mechanism, including gene transcription products like long non-coding RNA (lncRNA), competitively combine with microRNA (miRNA) via sponge adsorption [8]. After that, circRNA is provided with miRNA adsorption to modulate OS [9]. Circ RNA has also been reported to have a role in tumor chemo-resistance via the ceRNA mechanism. According to Wang et al, hsa_circ_0092276 facilitates the resistance of DXR to breast cancer (BC) via modulating the miR-348/ATG7 axis [10]. In addition, circ RNA has been confirmed to modulate the resistance of OS cells to cisplatin or DOX via the ceRNA mechanism. CircRNA ankyrin repeat and IBR domain containing 1 (ANKIB1) (circANKIB1; circRNA ID: has_circ_0009112, position: chr7:91,972,337–91,981,956) is a product of ANKIB1 mRNA splicing (http://www.circbank.cn/infoCirc.html?id=hsa_circANKIB1_024). Zhu *et al*. [11,12] state that circ_ANKIB1 is elevated in OS, and Yi-xin Du *et al*. maintain that circ_ANKIB1 accelerates the development and invasion of OS cells via modulating the miR-19b/SOCS3/STAT pathway [13]. However, the action and mechanism of circ_ANKIB1 in OS chemo-resistance were not reported.

MiRNAs are another non-coding RNA that modulates various tumor cells’ biological functions, involving cell differentiation and advancement [14]. MiRNAs mediate the chemotherapy resistance of OS, offering a promising therapeutic target [15–17]. MiR-26b has been reported as a tumor suppressor gene against OS [11,18], but its function in chemotherapy resistance has not yet been elucidated. Enhancer of zeste homolog 2 (EZH2) is a member of the histone methyltransferase family, which has a role in the carcinogenesis and chemotherapy resistance of OS [11,19].

The present investigation hypothesized that c**ircular RNA_ANKIB1 accelerates osteosarcoma chemo-resistance via binding microRNA-26b-5p and modulating** EZH2. This study aimed to determine the expressions of Circ_ANKIB1, EZH2, and **miRNA-26b-5p in OS**, determine the effects of circ_ANKIB1 knockdown on OS and investigate whether circ_ANKIB1 is a target of miR-26b-5p. Finally, the effects of **miRNA-26b-5p inhibition on** circ_ANKIB1 and **circ_ANKIB1 knockdown on DXR-resistant OS cells in *vivo were investigated***

## 2 Methods

### 2.1 Source and grouping of tissue specimens

Osteosarcoma biopsy samples were collected in Huai’an Second People’s Hospital from August 2017 to May 2020 and prepared through histopathology and surgical resection. In total, 61 OS specimens and 61 para-cancerous tissue specimens were collected. The samples were taken from patients who had not done radiotherapy or chemotherapy prior to enrollment; All the enrolled patients received DXR-based chemotherapy, and the patients were divided into the DXR-sensitive (n = 33) and the DXR-resistant (n = 28) groups, in line with the Huvos scoring system. The study was reviewed and authorized by the ethics committee of Huai’an Second People’s Hospital (Approval number: H1703211c). Informed written consent was obtained from all the participants prior to inclusion into the study.

### 2.2 Cell culture

Normal osteoblast cell hFOB1.19 and human KHOS, U2-OS OS cell lines were purchased from American Type Culture Collection (ATCC, Manassas, VA, USA) and used for the study. The hFOB1.19 cells were grown in a culture medium of a 1:1 mixture of Dulbecco’s Modified Eagle’s Medium without phenol red and Ham’s F12 medium, augmented with 2.5 mM of l-glutamine, 0.3 mg/mL of G418, (all from Sigma) penicillin(100 U/ml) and streptomycin (100 U/ml). (Thermo Fisher Scientific, Waltham, MA), and 10% of fetal bovine serum, FBS (Gibco, Waltham, MA, USA) in a humidified 5% CO2 atmosphere at a temperature of 37°C.

The U2-OS and KHOS cells were cultured in Dulbecco’s modified Eagle medium (DMEM) supplemented with 10% fetal bovine serum (FBS)(Gibco, Uruguay), penicillin(100 U/ml), and streptomycin (100 U/ml) (Thermo Fisher Scientific, Waltham, MA). The cells were cultured at 37°C and 5% CO_2_. DXR resistant cell line KHOS/DXR and U2OS/DXR were generated via exposing the parental cell lines KHOS and U2OS to increasing doses of DXR (Sigma, San Francisco, CA, USA). The DXR resistant cell lines culture was done in the same DMEM culture medium with 1 µg/mL DXR to sustain their resistant phenotype.

### 2.3 Cell transfection

Cells were seeded in a 6-well plate, and the relevant sequences were transfected separately. The cells were divided into 6-well plates one day before transfection to obtain a cell density of about 70% during transfection. Transfection was done using lipofectamine 2000 reagent Kit (11,668–027, Invitrogen, Carlsbad, CA, USA) according to the manufacturer’s guidelines. Furthermore, the si-circ_ANKIB1#1/-circ_ANKIB1#2, si/mimic/inhibitor-NC, miR-26b-5p inhibitor/mimic, and Oe-EZH2 were prepared, synthesized, and transfected into the cells. All vector construction, plasmids, identification of sequencing, virus packaging, and detection of titers were provided by the Shanghai Genechem Co., Ltd. (Shanghai, China).

### 2.4 Reverse transcription-quantitative polymerase chain reaction (RT-qPCR)

Total RNA in tissues and cells was extracted and measured using TRIzol reagent (Invitrogen) according to the manufacturer’s instructions. RNA was reverse transcribed into cDNA using the reverse transcription kit (K1621, Fermentas, Maryland, NY, USA). Each gene was tested with a fluorescence quantitative polymerase chain reaction (PCR) kit (Takara, Dalian, China) and examined with a real-time fluorescent quantitative PCR instrument (Thermo Fisher Scientific, Massachusetts, USA). U6 was used as the internal reference for miR-26b-5p, while glyceraldehyde-3-phosphate dehydrogenase (GAPDH) was utilized as the internal reference for circ_ANKIB1 and EZH2 [20]. Calculation of the target gene was done with the 2^−ΔΔCt^ method, N = 3, and the average was taken. Primer sequences of circ_ANKIB1, miR-26b-5p, and EZH2 were designed as shown in Table 1.

**Table 1.** Primer sequences

Genes	Primer sequences (5’ – 3’)
MiR-26b-5p	F: 5’-CCTGTGGAGATTGATGGGGT-3’
	R: 5’-TCTCTGGGCCTCTGACATTC-3’
Circ_ANKIB1	F: 5’-AGACCGCAGACATGCTCC-3’
	R: 5’-AGTCCCTAATATCCTATTCATTCCA-3’
EZH2	F: 5ʹ-GTACACGGGGATAGAGAATGTGG-3′
	R: 5ʹ-GGTGGGCGGCTTTCTTTATCA-3ʹ
U6	F: 5ʹ-CTCGCTTCGGCAGCACA-3′
	R: 5′-AACGCTTCACGAATTTGCGT-3′
GAPDH	F: 5ʹ-GGGAGCCAAAAGGGTCAT-3ʹ
	R: 5ʹ-GAGTCCTTCCACGATACCAA-3ʹ

Note: F: Forward; R: Reverse

### 2.5 RNase R treatment

The RNAs (1 µg) were treated from cells using RNase R (2 U/µg, GeneSeed, Guangzhou, China); Incubation and inactivation were performed. Reverse transcription of the treated RNAs was done with divergent or convergent primers, which were tested. N = 3, and the average was taken.

### 2.6 RNA immunoprecipitation (RIP)

RNA immunoprecipitation (RIP) was done as described elsewhere [21]. Determination of the RIP was done with a Magna RIP Kit (Millipore, Billerica, MA, USA). To this end, collection and resuspension of 1 × 10^7^ OS cells were performed in 100 μL of RIP lysis buffer plus protease inhibitor cocktail and RNase inhibitors. Approximately 200 μL of the cell lysates were then incubated with 5 μg magnetic beads conjugated to anti-Argonaute 2 (AGO2) antibody (Millipore) or control rabbit IgG (Millipore). After digestion with proteinase K, extraction of the immunoprecipitated RNAs was performed. Test of enrichment of circ_ANKIB1and miR-26b-5p was finally conducted. The experiment was done in triplicate.

### 2.7 RNA-pull down

RNA-pull down was done as described elsewhere [21]. Transfection of the cells was done using 50 nM biotin-labeled WT-bio-miR-26b-5p and MUT-bio-miR-26b-5p (Wuhan GeneCreate Biological Engineering Co., Ltd., Wuhan, China). The cells were then collected and incubated for 10 min in a specific lysis buffer (Ambion, Austin, Texas, USA). The lysates were incubated with M-280 streptavidin magnetic beads (S3762, Sigma, USA) pre-coated with RNase-free and yeast tRNA (TRNABAK-RO, Sigma, USA). The samples were then rinsed with pre-cooled lysis buffer, low/high-salt buffer. The combined RNA was purified and examined for the enrichment of the circ_ANKIB1. N = 3, and the average was taken.

### 2.8 The luciferase activity assay

Prediction of the targeting sites of miR-26b-5p and circ_ANKIB1/EZH2 was performed as previously described [22]. Circ_ANKIB1/EZH2-3’-untranslated region (3ʹUTR) sequence primer was designed, and mutation of the target sites of its 3ʹUTR sequence primer miR-26b-5p was used to gain the 3ʹUTR MUT fragment. To attain the same sticky ends, the pMIR-Report vector plasmid and the target gene were simultaneously digested via Hind III and Mlu I restriction enzyme. The T4 ligase was used to transform the target gene and the pMIR-Report vector plasmid into the Escherichia coli JM109 strain. After the advancement of a single colony, colony PCR and recombinant plasmids were digested and tested, and sequencing was performed. The miR-26b-5p mimic and its negative control (all designed and synthesized via GenePharma Co. Ltd. Company, Shanghai, China) were separately transfected into the cells with circ_ANKIB1/EZH2-3ʹUTR WT and circ_ANKIB1/EZH2-3ʹUTR MUT. After the development of the cells with a specific density, collection and lysis of the cells were conducted. Examination of the luciferase activity was done with a luciferase detection kit (Promega, USA) and an ultra-micro ultraviolet photometer (Bio-Rad, USA). N = 3, and the average was taken.

### 2.9 Western blot

The cells were transferred in a centrifuge tube, 100 μL radio-immunoprecipitation assay lysis solution (R0020, Beijing solarbio science & technology co. ltd., Beijing, China) was added and homogenized at 3000 rpm. The protein concentration was determined using bicinchoninic acid (BCA) kit (AR0146, Wuhan Boster Company, Wuhan, China), and the concentration of each sample was adjusted to 3 μg/μL.

The extracted protein was loaded into the loading buffer, boiled for 10 min, and a 30 μg sample was loaded per well, and the protein was separated using 10% polyacrylamide gel electrophoresis. The protein was transferred to polyvinylidene fluoride (PVDF) membrane (P2438, Sigma, USA) via semi-dry electroporation transfer method, blocked with 5% bovine serum albumin (10-L16, Beijing Zhongsheng Likang Technology Co., Ltd., China), and the primary antibody was then added at; EZH2 (1: 1000, Abcam, Cambridge, MA, USA), P-gp (1: 500, Abcam, Cambridge, MA, USA), multidrug resistance protein 1 (MRP1) (1: 1000, Abcam, Cambridge, MA, USA) and lipoprotein receptor-related protein (LRP) (1: 1000, Abcam, Cambridge, MA, USA).

Later, corresponding horseradish peroxidase-labeled IgG (1: 1000, Wuhan Boster Company, Wuhan, China) was added and incubated. The membrane was then rinsed, and a band was developed using a chemiluminescence reagent. The GADPH (ab181602, Abcam, USA, 1: 10,000) was used as the internal reference. The band’s development was analyzed with Bio-rad Gel Dol EZ imager (GEL DOC EZ IMAGER, Bio-rad, California, USA). A gray value analysis of the target band was done via Image J software (National Institutes of Health, Bethesda, MD, USA). The experiment was done in triplicates.

### 2.10 Cell-counting kit-8 (CCK-8)

The cells were harvested at the logarithmic growth phase, and the pellets were collected via conventional digestion and centrifugation. Cells were then resuspended, counted, diluted, and adjusted to a 1 × 104/m concentration. Approximately 200 μL of the diluted cell suspension was added into 96-well plates and incubated. After adherence, the cells were treated with gradient concentrations of DXR. The cells were then cultured for 48 h, and the old medium was then discarded and replaced with 10% CCK-8 fresh medium as per the manufacturer’s instructions of Cell Counting Kit-8 (Dojindo, Tokyo, Japan). The cells were then incubated for 3 h, and the absorbance value was examined at 450 nm. The repression rate of the cell proliferation = (1-the experiment A /the control A) × 100%, and IC50 of the cells lines was tested which was defined as a chemotherapeutic drug concentration proving the successful construction of drug-resistant strains, and repressed the cell proliferation with 50%. N = 3, and the average was taken.

### 2.11 Colony formation experiment

A colony formation assay was done as previously described [23]. The cells at the logarithmic growth phase were harvested, and the cell pellets were collected through conventional digestion and centrifugation. The cell pellets were resuspended and counted, and the concentration was adjusted to 1 × 105/mL and diluted with 4 ml of culture medium in 6-well plates. The cells were incubated for 3 weeks to the point of the development of clones. The cells were fixed in methanol for 15 minutes, and the number of visible clones was counted after crystal violet staining. The experiment was done in triplicates.

### 2.12 Flow cytometry

The flow cytometry was done as previously described [24]. Cells in the logarithmic phase were detached and collected in a flow tube with 0.25% pancreatin (without ethylene diamine tetraacetic acid (EDTA)) (PYG0107, Boster, Wuhan, China). The cells were then centrifuged, and supernatants were collected and washed twice. The cells were then stained with Annexin-V-fluorescein isothiocyanate (FITC), propidium iodide (PI), and 4-(2-hydroxyethyl)-1-piperazineëthanesulfonic acid (HEPES) buffer solutions according to the instructions of Annexin-V-FITC Cell Apoptosis Detection Kit (K201-100, Biovision, USA). Approximately 1 × 10^6^ cells were resuspended in 100 µL staining solution, incubated, and 1 mL of HEPES buffer solution was finally added. FITC and PI fluorescence was analyzed via exciting 515 nm 620 nm bandpass filters at 488 nm. The cell apoptosis was assessed via flow cytometry (BD Biosciences, Franklin Lakes, NJ, USA). The assay was repeated three times.

### 2.13 Scratch test

Scratch assay was done as previously described [25]. The cells were seeded in a 6-well plate at 4 × 10^5^ cells/well. After transfection, at a confluence of around 80%, a straight line was made along the central axis at an angle perpendicular to the bottom of the plate surface using a sterile 10 μL pipette tip. The serum-free medium in the culture was replaced and cultured further for 0.5 h. Cell migration was observed, and the images were captured using an inverted optical microscope at 0 and 24 h. the assay was done in triplicate.

### 2.14 Transwell

Transwell assay was done as previously described [26]. The cell invasion in each group was examined using a Matrigel-coated Transwell chamber (Becton Dickinson). The cells at the logarithmic growth phase were harvested, transfected, resuspended, and counted. Approximately 1 × 10^5^ of cells (200 μL) was added in the upper chamber of Transwell, and 500 μLof 1 Roswell Park Memorial Institute 1640 medium supplemented with 20% FBS was added in the lower chamber of Transwell. Cells in the Transwell chamber were collected, and the cells adjacent to the lateral membrane of the upper chamber were discarded. The cells were fixed with 4% paraformaldehyde solution, stained with crystal violet. The cells were observed, photographed under an optical microscope, and analyzed using ImageJ software (National Institutes of Health, Bethesda, MD, USA). The experiment was done in triplicates.

### 2.15 Subcutaneous tumorigenesis in nude mice

BALB/c nude mice (4–6 weeks) were purchased from Shanghai Slack Company, Shanghai, China. The mice were bred under specific pathogen-free (SPF) environmental conditions. The animal experiment was authorized by the committee on Anima Care and Use of Huai’an Second People’s Hospital.

Transfection of KHOS/DXR and U2OS/DXR cells was done after si-NC/circ_ANKIB1#1. The single-cell suspension (2 × 10^6^ pcs/mL) was subcutaneously injected into the back of nude mice with a disposable sterile syringe to study the effect of circ_ANKIB1 on OS drug-resistant cells in *vivo*. In addition, 5 mice were used in each group. Behind a week, the mice were injected with 2 mg/kg DXR (Solarbio) 7 days after the introduction of the tumor. The volume of the tumor was monitored once a week. The mice were sacrificed after 4 weeks, and the tumor was harvested. The tumor weight and dimensions were then measured. The tumor volume was calculated using the formula; Tumor volume = 0.5 × (long axis × short axis) ^2^.

### 2.16 Statistical analysis

Data analysis was done using GraphPad Prism 8. The data were presented as mean ± standard deviation (SD). The Two-group comparison was done via t-test. The multiple groups’ comparison was made using one-way analysis of variance (ANOVA), and pairwise comparison was determined using Tukey’s multiple comparisons test. Analysis of the relevance of circ-ANKIB1 in OS patients’ clinic-pathological features was also done and. Pearson test was used to carry out the correlation analysis. *P* < 0.05 was regarded as significant.

## 3 Results

### 3.1 Circ_ANKIB1 and EZH2 are upregulated in OS tissues

Osteosarcoma is a common bone malignancy in children and adolescents. Chemotherapeutic drug resistance is the primary factor impacting the surgical outcome and prognosis of patients with osteosarcoma. The present investigation hypothesized that circular RNA_ANKIB1 accelerates osteosarcoma chemo-resistance via binding microRNA-26b-5p and modulating EZH2. The study aimed to determine the expressions of circ_ANKIB1, EZH2, and miRNA-26b-5p in OS, determine the effects of circ_ANKIB1 knockdown on OS and investigate whether circ_ANKIB1 is a target of miR-26b-5p. Finally, the effects of miRNA-26b-5p inhibition on circ_ANKIB1 and circ_ANKIB1 knockdown on DXR-resistant OS cells in *vivo* were investigated.

**Figure 1.**
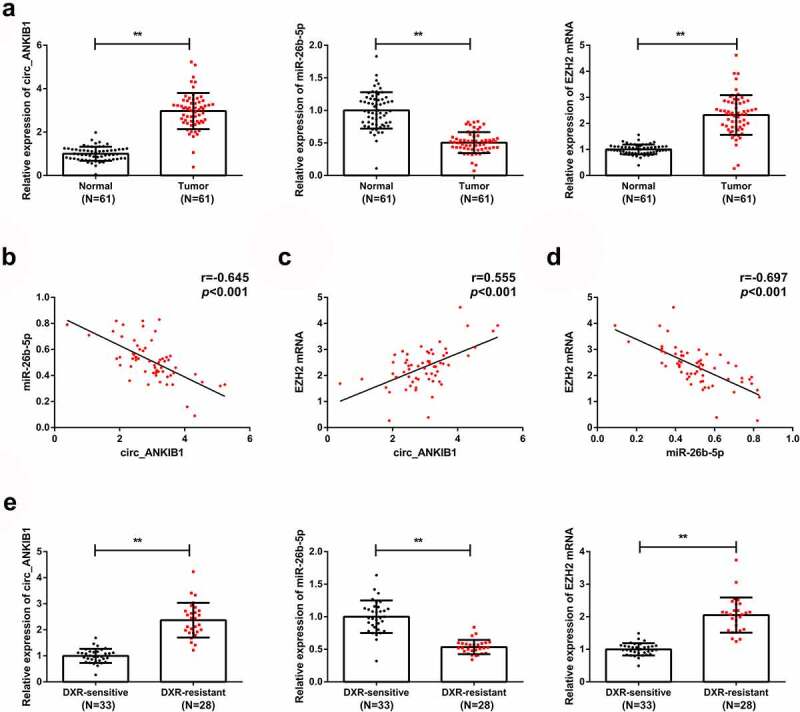
Circ_ANKIB1 and EZH2 are upregulated in OS tissues.

The circ_ANKIB1, miR-26b-5p, and EZH2 expression in OS tissues and corresponding para-cancerous tissues were determined, and the results confirmed an increased circ_ANKIB1 expression in the tumor compared to the normal tissues. However, miR-26b-5p expression was reduced in OS tissues compared to the para-cancerous tissues. EZH2 expression was also elevated in the OS tissues compared to the normal tissues ((Figure 1a). The analysis of the correlation of circ_ANKIB1, miR-26b-5p with EZH2 in patients with OS showed that miR-26b-5p is negatively correlated with circ_ANKIB1 (Figure 1b) while circ_ANKIB1 and EZH2 were positively associated (Figure 1c). EZH2 and MiR-26b-5p demonstrated a negative correlation (Figure 1d). Additionally, analysis of circ_ANKIB1 and clinic-pathological features of OS patients clarified that: Tumor node metastasis (TNM) grade, differentiation, and tumor size of patients with OS were positively correlated with circ_ANKIB1, as proved in Table 2.

**Table 2.** Association of relative circ_ANKIB1 and OS patients’ clinic-pathological features

	n	**Circ_ANKIB1**		
Clinicopathological data	61	The declined (n = 30)	The elevated (n = 31)	*P*
Age (years)				0.714
Less than 60	36	17	19	
60 or more	25	13	12	
Gender				0.363
Male	28	12	16	
Female	33	18	15	
TNM staging				0.029
I + II	29	10	19	
III + IV	32	20	12	
Tumor diameter				0.015
Less than 5 cm	27	18	9	
5 cm or more	34	12	22	
Lymph node metastasis				0.006
Yes	37	13	24	
No	24	17	7	

The data in this table were enumeration data, and the test was with Chi-square.

A: Detection of circ_ANKIB1, miR-26b-5p, EZH2 in OS tissue and para-cancerous tissues was via RT-qPCR; B: Correlation analysis of circ_ANKIB1 and miR-26b-5p in the OS tissue was performed; C: Associated analysis of circ_ANKIB1 and EZH2 in OS tissue was performed; D: Correlation analysis of miR-26b-5p and EZH2 in OS tissue was conducted; E: Assessment of circ_ANKIB1, miR-26b-5p, EZH2 in DXR-resistant OS tissue and DXR-sensitive OS tissues was done through RT-qPCR; *P < 0.05, **P < 0.01. Analysis of the association of circ-ANKIB1 with the clinic-pathological features of OS patients was exerted, and the correlated analysis was performed with the Pearson test.

RT-qPCR was then used to determine the circ_ANKIB1, miR-26b-5p, and EZH2 mRNA expressions in DXR-resistant and DXR-sensitive OS tissues. The observations demonstrated increased circ_ANKIB1 and EZH2 mRNA expressions but reduced miR-26b-5p mRNA expressions in DXR-resistant OS tissues compared to the DXR-sensitive OS tissues (Figure 1e).

### 3.2 Circ_ANKIB1, EZH2, and miR-26b-5p expressions in DXR-resistant OS tissue

**Figure 2.**
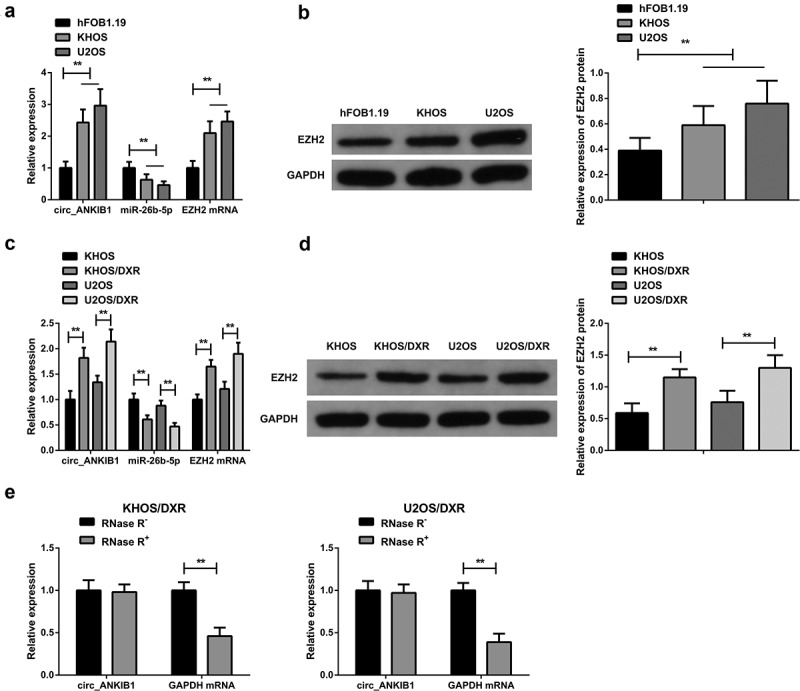
Increased circ_ANKIB1, EZH2, but suppressed miR-26b-5p expressions in DXR-resistant OS tissue.

The hFOB1.19, KHOS, and U2OS cells were cultured, and DXR resistant cell lines KHOS/DXR and U2OS/DXR were constructed. RT-qPCR and western blotting were used to determine the mRNA expressions of MiR-26b-5p, circ_ANKIB1, and EZH2. According to the results, MiR-26b-5p expression was significantly reduced while circ_ANKIB1 and EZH2 expressions were elevated in KHOS and U2OS tumor cell lines compared to the hFOB1.19 normal cell line (Figure 2a, b). Additionally, miR-26b-5p expression in the drug-resistant cell lines KHOS/DXR and U2OS/DXR was significantly reduced compared to the parental lines, but circ_ANKIB1 and EZH2 expressions were significantly increased (Figure 2c, d). Additionally, circ_ANKIB1 was resistant to RNase R treatment, while digestion of GAPDH was via RNase R, clarifying that circ_ANKIB1 has a ring structure (Figure 2e).

A: Investigation of circ_ANKIB1, miR-26b-5p, EZH2 in normal osteoblast cell hFOB1.19 and OS cell lines KHOS, U2OS was done using RT-qPCR; B: Examination of EZH2 protein in Normal osteoblast cell hFOB1.19 and OS cell lines KHOS, U2OS was via WB; C: Test of circ_ANKIB1, miR-26b-5p, EZH2 in the cell lines KHOS, U2OS and DXR resistant cell lines KHOS/DXR, U2OS/DXR was via RT-qPCR; D: Examination of EZH2 protein in cell lines KHOS, U2OS, and DXR resistant cell lines KHOS/DXR, U2OS/DXR was via WB; E: Determination of the circular (circ) features of circ_ANKIB1 was via RNase R treatment; *P < 0.05, **P < 0.01. N = 3.

## 3.3 circ_ANKIB1 knockdown suppresses DXR-resistant OS cells

**Figure 3.**
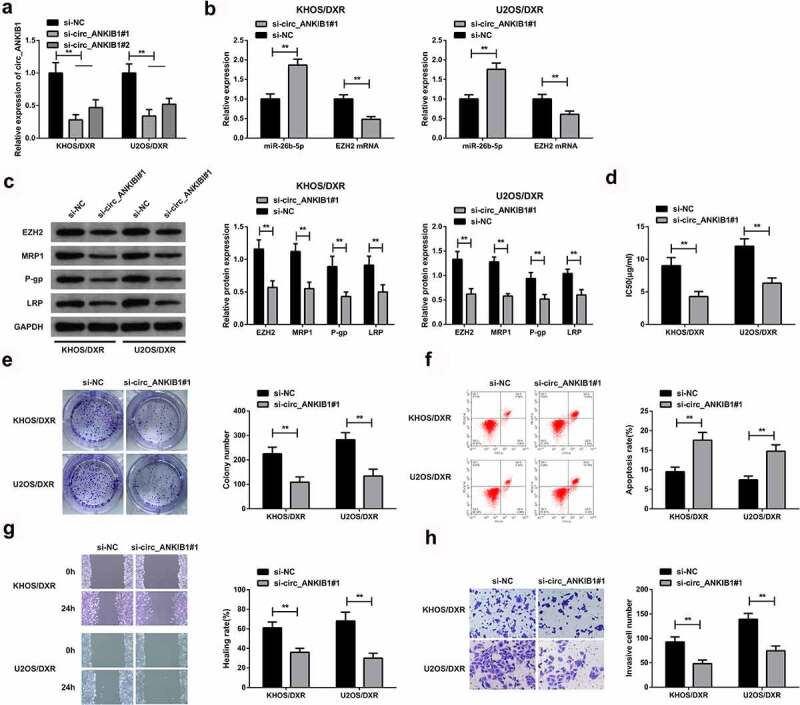
Circ_ANKIB1 knockdown suppresses DXR-resistant OS cells.

The KHOS/DXR and U2OS/DXR cells were transfected with si-circ_ANKIB1#1 and si-circ_ANKIB1#2 or the si-NC, and then si-circ_ANKIB1#1 mRNA expression was determined using RT-qPCR. The results demonstrated a significantly reduced si-circ_ANKIB1#1 expression (Figure 3a). Further, the expression of miR-26b-5p and EZH2 were assessed in the KHOS/DXR and U2OS/DXR cells transfected with circ_ANKIB1#1 or si-NC. The results confirmed an increased miR-26b-5p mRNA expression but a reduced EZHZ mRNA expression in KHOS/DXR and U2OS/DXR cells transfected with circ_ANKIB1#1 compared to the controls (Figure 3b). The EZHZ, MRP-1, P-gp, LRP expressions were determined in the si-circ_ANKIB1 or the si-NC cells. The results confirmed significantly reduced EZHZMRP1, P-gp, and LRP protein expressions in the si-circ ANKIB1-transfected KHOS/DXR and U2OS/DXR cells compared to the si-NC transfected cells (Figure 3b). Knockdown of circ_ANKIB1 significantly reduced the IC50 for the KHOS/DXR and U2OS/DXR cells. In addition, the clone formation was significantly reduced in the cells transfected with si- circ_ANKIB1 compared to the si-NC-transfected cells (Figure 3e). Knockdown of circ_ANKIB1 also significantly increased the apoptosis of KHOS/DXR and U2OS/DXR cells compared to the si-NC transfected cells (figure 3f). Finally, the wound healing and cell invasion capacity were significantly reduced following transfection of the cells with si-circ ANKIB1 compared to transfection with si-NC accelerated the cell advancement. (Figure 3g-h). These observations confirmed that circ_ANKIB1 silencing effectively suppressed DXR-resistant OS cells.

A: Test of the cells circ_ANKIB1 after transfection was via RT-qPCR; B: Detection of the cells miR-26b-5p, EZH2 after transfection was via RT-qPCR; C: Test of the cells EZH2, MRP1, P-gp, and LRP protein after transfection was via WB; D: Examination of IC50 after transfection was via CCK8 assay; E: Assessment of the cell clone formation after transfection was done via plate cloning experiment; F: Examination of the cell apoptosis after transfection via flow cytometry; G: Test of the cell migration after transfection was done via scratch assay; H: Examination of the cell invasion after transfection was done through Transwell assay; *P < 0.05, **P < 0.01. N = 3.

## 3.4 circ_ANKIB1 is a target of miR-26b-5p in OS

**Figure 4.**
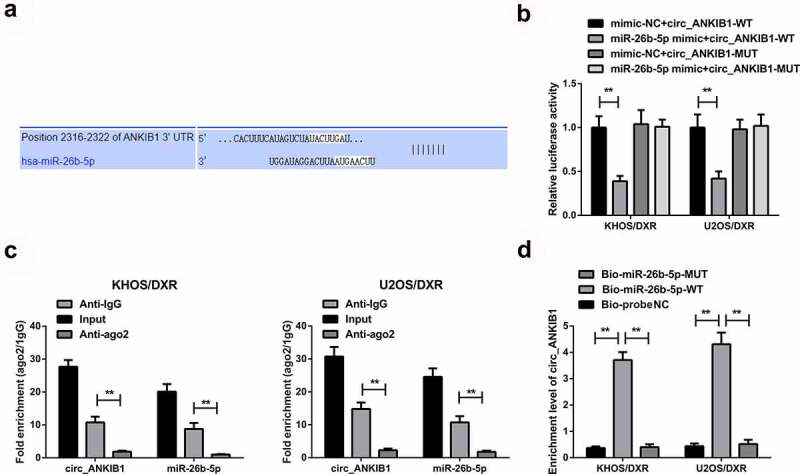
Circ_ANKIB1 is a target of miR-26b-5p in OS.

We then investigated whether circ_ANKIB1 inhibits DXR-resistant OS cells by targeting miR-26b-5p. Analysis of miR-26b-5p target gene using Pictar, miRanda, and Targetscan prediction tools showed that circ_ANKIB1 binds miR-26b-5p in the 3ʹUTR region, as shown in Figure 4a. In addition, the luciferase activity was significantly reduced in cells co-transfected with miR-26 mimic and wild-type circ_ANKIB1compared to the mimic-NC+ wild-type circ_ANKIB1. However, no significant differences were observed in the luciferase activity of mutant 3ʹUTR versus the mimic-NC, confirming that Circ_ANKIB1 specifically binds with miR-26b-5p, as shown in Figure 4b. Circ_ANKIB1 and miR-26b-5p enrichment in Anti-AGO2 pellets were significantly increased compared to the Anti-IgG, as presented in Figure 4c. The enrichment of Circ_ANKIB1 in the Bio-miR-5-WT was remarkably elevated compared to the abundance of Circ_ANKIB1 in the Bio-miR-5-MUT or the Bio-probe NC, as presented in Figure 4d.

A: Predicting the binding site of circ_ANKIB1 and miR-26b-5p was done using Pictar, miRanda, and Targetscan prediction tools; B: Verification of the combination of circ_ANKIB1 and miR-26b-5p was carried out by the dual-luciferase; C: Test of endogenous association of miR-26b-5p with circ_ANKIB1 via RIP; D: Examination of the enrichment of miR-26b-5p for circ_ANKIB1 was via RNA pull-down; *P < 0.05, **P < 0.01. N = 3.

### 3.5 MiR-26b-5p inhibition reverses the suppressive effect of circ_ANKIB1 in OS

**Figure 5.**
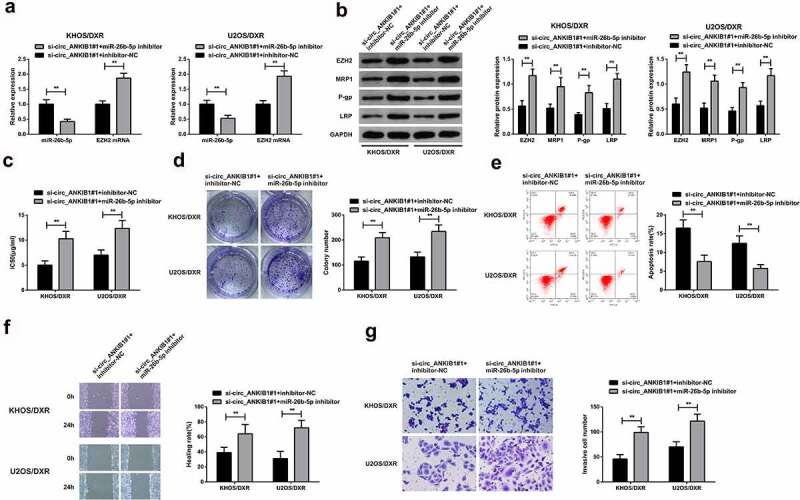
MiR-26b-5p inhibition reverses the suppressive effect of circ_ANKIB1 against DXR-resistant.

We then aimed at assessing the role of miR-26b-5p inhibitors in the control of OS. To this effect, the KHOS/DXR and U2OS/DXT cells were con-transfected with si-circ_ANKIB1#1+ miR-26b-5p inhibitor or si-circ_ANKIB1#1 and inhibitor-NC. The expression of miR-26b-5p and EZH2 mRNA were determined through RT-qPCR. According to the observations, miR-26b-5p mRNA expression was significantly reduced in the si-circ_ANKIB1#1+ inhibitor-NC compared to the si-circ_ANKIB1#1+ miR-26b-5p inhibitor. However, EZH2 mRNA expression was significantly increased in the circ_ANKIB1#1+ miR-26b-5p inhibitor compared to the si-circ_ANKIB1#1+ inhibitor-NC, as shown in Figure 5a. Determination of proteins expression using western blotting confirmed a significant elevation of EZH2, MRP1, P-gp, and LRP proteins in the cells co-transfected with si-circ_ANKIB1#1 miR-26b-5p inhibitor compared to the cells co-transfected with si-circ_ANKIB1#1 and inhibitor-NC, as shown in Figure 5b.

A: Testing miR-26b-5p and EZH2 were done by RT-qPCR; B: Detection of EZH2, MRP1, P-gp, and LRP protein was carried out through WB; C: Test of the cell IC50 via CCK8 experiment; D: Examining the cell colony formation was via plate cloning experiment; E: Test of the cell apoptosis was via flow cytometry; F: Examination of cell migration was done via scratch experiment; G: Test of cell invasion was done using Transwell assay; In the cells after transfection; *P < 0.05, **P < 0.01. N = 3.

.The IC50 and colony numbers were also significantly increased in the cells co-transfected with si-circ_ANKIB1#1 and miR-26b-5p inhibitor compared to those co-transfected with si-circ_ANKIB1#1 and inhibitor-NC, as shown in Figure 5c and 5D. However, apoptosis was significantly reduced in the cells co-transfected with si-circ_ANKIB1#1 and miR-26b-5p inhibitor compared to the cells co-transfected with si-circ_ANKIB1#1 and inhibitor-NC, as shown in Figure 5e. Finally, the wound healing and cell invasion ability were also significantly increased in the cells co-transfected with si-circ_ANKIB1#1 and miR-26b-5p inhibitor compared to the cells co-transfected with si-circ_ANKIB1#1 and inhibitor-NC, as shown in figure 5f and 5g. These observations confirmed that inhibition of miR-26b-5p reverses the protective role of circ_ANKIB1#1 silencing against OS.

### 3.6 MiR-26b-5p targets EZH2 to repress DXR-resistant OS cells

**Figure 6.**
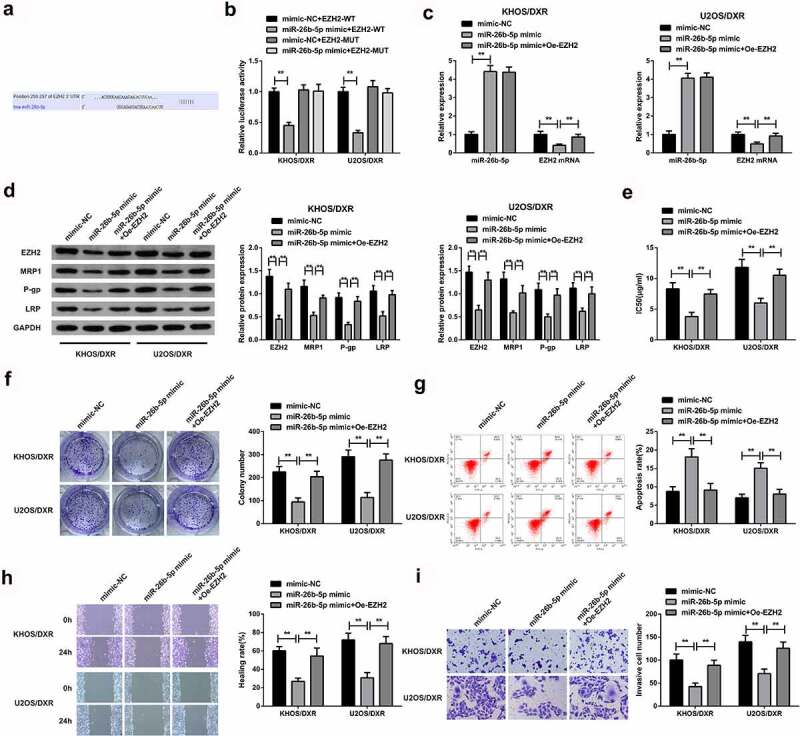
MiR-26b-5p targets EZH2 to repress DXR-resistant OS cells.

The downstream mechanism of miR-26B-5p was then investigated. According to the gene target prediction result, EZH2 was the target gene of miR-26b-5p (Figure 6a). The luciferase binding activity assay confirmed a significantly reduced activity in the cells co-transfected with miR-26b-5p mimic and wild-type EZH2 compared to cells co-transfected with mutant 3ʹUTR or the mimic-NC, clarifying that miR-26b-5p targeted EZH2, as shown in Figure 6b. The cells were then transfected with MiR-26b-5p-mimic, MiR-26b-5p mimic+ Oe-EZHZ or mimic-NC, miR-26b-5p, and EZH2 mRNA expression was then determined through RT-qPCR.

A: Predicting the biding sites of miR-26b-5p and EZH2 was done using Pictar, miRanda, and Targetscan prediction tools; B: Determination of the targeting of miR-26b-5p and EZH2 was via the dual-luciferase; C: Test of miR-26b-5p with EZH2 was via RT-qPCR; D: Examination of EZH2, MRP1, P-gp, and LRP protein was via WB; E: Test of IC50 was via CCK8 assay; F: Examination of the cell colony formation was via plate cloning experiment; G: Test of the cell apoptosis was via flow cytometry; H: Examination of cell migration was via scratch assay; I: Test of cell invasion was via Transwell assay; Figure C-I, in the cells behind transfection. *P < 0.05, **P < 0.01. N = 3.

The results confirmed a significant elevation of miR-26b-5p mRNA expression but a significant decrease of EZH2 mRNA in the miR-26b-5p-mimic and MiR-26b-5p mimic+ Oe-EZHZ group compared to the mimic-NC, as shown in Figure 6c. Furthermore, miR-26b-5p, EZH2 and MRP1, P-gp, and LRP protein were remarkably inhibited in the miR-26b-5p-mimic and MiR-26b-5p mimic+ Oe-EZHZ group compared to the mimic-NC, as proved in Figure 6d. The IC50 and colony numbers were significantly reduced in the miR-26b-5p-mimic and miR-26b-5p mimic+ Oe-EZHZ group compared to the mimic-NC (Figure 6e-6F). The apoptosis was significantly increased in the miR-26b-5p mimic compared to the miR-26b-5p mimic+ Oe-EZHZ or the mimic-NC group (Figure 6g). Finally, the wound healing and cell invasion capacity was significantly increased in the miR-26b-5p mimic+ Oe-EZHZ compared to the miR-26b-5p mimic group (Figure 6h and 6I). Taken together, these observations confirmed that miR-26b-5p targets EZH2 to repress DXR-resistant OS cells.

### 3.7 *Knockdown of circ_ANKIB1 suppresses DXR-resistant OS cells in* vivo

**Figure 7.**
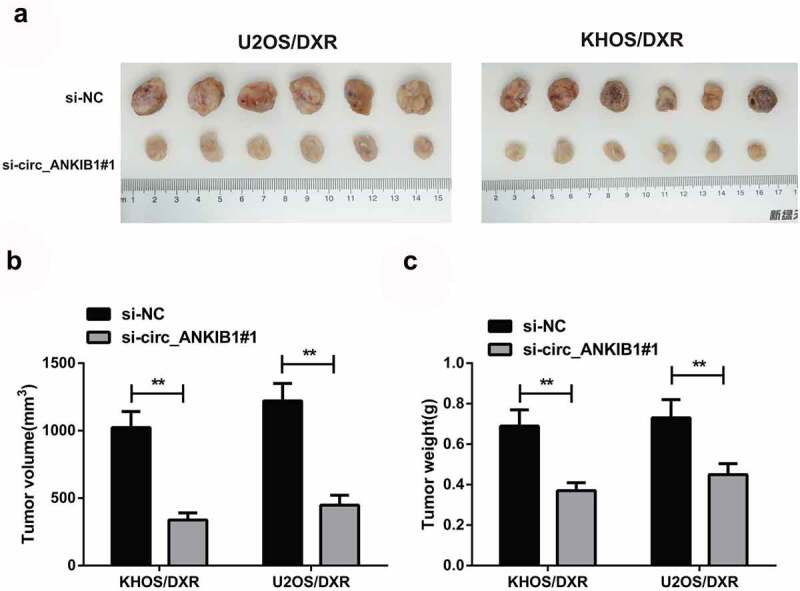
Knockdown of circ_ANKIB1 suppresses DXR-resistant OS cells in *vivo.*

We finally investigated the effects of circ_ANKIB1 knockdown in the progression of OS in vivo. The xenograft tumor mouse model was constructed to determine the function of circ_ANKIB1 on DXR drug-resistance in *vivo*. The results confirmed that knockdown of circ_ANKIB1 significantly suppressed the tumor volume and weight of the tumor mice model (Figure 7a-c). These results together showed that the knockdown of circ_ANKIB1 repressed DXR-resistant OS cells.

A: Representative tumor images of transplanted tumors in nude mice of each group (n = 6); B: Comparison of transplanted tumor volume of nude mice in each group; C: Comparison of transplanted tumor weight of nude mice in each group; *P < 0.05, **P < 0.01. N = 5.

## 4 Discussion

Resistance to chemotherapy is always the major challenge in OS treatment [11,27]. DXR is among the most effective drugs in standard chemotherapy for OS, but 40–45% of patients with advanced OS do not respond or only partially respond to DXR [28,29] . Drug resistance in the tumor is a complex, multi-step process. Recently, with the advancement of bioinformatics analysis and elevated-throughput sequencing technology, the biological functions of circular RNA (circRNA) have been gradually identified [30]. Some studies have reported that circRNA has a role in developing resistance to chemotherapy in human cancers such as OS [31].

Consequently, targeting circRNA to arrest DXR resistance might be a promising treatment strategy. In this study, circ_ANKIB1was significantly elevated in OS tissues and cells. Circ_ANKIB1 in DXR-resistant OS tissues and cells was increased compared to the DXR-sensitive tissues and cells. Silencing of circ_ANKIB1 repressed the progression of DXR-resistant OS cells via miR-26b-5p /EZH2 axis.

According to recent studies, circRNA has a vital role in controlling OS chemo-resistance. For instance, Ji Y *et al*. maintain circ_0002060 strengthens Adriamycin resistance in OS via modulating miR-198/ABCB1 axis [32]. Additionally, circ_0001721 has been reported to heighten Adriamycin resistance via miR-758/TCF4 axis and facilitate tumorigenesis of OS [33]. Increased circ-LARP4 is also associated with reduced Enneking phase and prolonged survival and improved chemo-sensitivity of OS to cisplatin and Adriamycin via sponging miR-424 [34]. In the present study, circ_ANKIB1 is elevated in OS and linked with TNM grade, tumor size, and lymph node metastasis, which agrees with the previous findings [13]. The modulation of circ_ANKIB1 in OS chemo-resistance was further explored, which clarified thatcirc_ANKIB1 silencing repressed the progression of advancement DXR-resistant OS cell lines. Furthermore, this trend was also confirmed in the in *vivo* mouse experiments. As confirmed, circ_ANKIB1 has a crucial biological function in the chemo-resistance of OS. Furthermore, circ_ANKIB1 binds with miR-26b-5p. Circ_ANKIB1 had a role in the chemical resistance of OS via the competitive endogenous RNA mechanism (ceRNA mechanism)”.

MicroRNAs (miRNAs), small non-coding RNAs, control the gene, and the critical function of miRNAs within cancer pathogenesis and response to treatment has been confirmed in different cancers [35]. Previous studies have reported the repression of miR-26b-5p in different tumors such as lung adenocarcinoma LAC [36], Human Papillary Thyroid Cancer [37], and Burkitt lymphoma [38]. The results of the present study observed that miR-26b-5p was silenced in OS and DXR-resistant OS tissues and cells. Circ_ANKIB1 targeted adsorption of miR-26b-5p and miR-26b-5p inhibition reversed the effect of circ_ANKIB1 silencing on DXR resistance OS cells, which proved that circ_ANKIB1 modulated DXR resistant OS cells via miR-26b-5p binding. Elevated miR-26b-5p represses the DXR resistant OS cells, and miRNAs have a role in establishing and advancing cancer via interacting with the targeted genes [39]. In this study, EZH2 was elevated in DXR-resistant OS cells, consistent with the previous findings. Yuan J *et al*. [27]elucidate that EZH2 is elevated in DXR-resistant OS cells and targets miR-760 in arresting the progression of DXR-resistant OS cells. The results clarified that EZH2 was elevated in OS and DXR-resistant OS. Furthermore, miR-26b-5p inhibited DXR-resistant OS via targeting EZH2.

## 5 Conclusion

In conclusion, circ_ANKIB1 accelerates OS resistance to DXR via modulating miR-26b-5p and EZH2. This study provides a novel understanding of the biological mechanism of DXR resistance in OS, highlighting a possible target approach for developing an alternative therapeutic intervention against osteosarcoma.
